# LLLT accelerates experimental wound healing under microgravity conditions via PI3K/AKT-CCR2 signal axis

**DOI:** 10.3389/fbioe.2024.1387474

**Published:** 2024-08-13

**Authors:** Rongan Ye, Yu He, Wei Ni, Yiqiu Zhang, Ying Zhu, Muqing Cao, Ruida He, Min Yao

**Affiliations:** ^1^ Department of Plastic and Reconstructive Surgery, Shanghai Ninth People’s Hospital, Shanghai Jiao Tong University, School of Medicine, Shanghai, China; ^2^ Shanghai Key Laboratory of Reproductive Medicine, Department of Histoembryology, Genetics and Developmental Biology, Shanghai Jiao Tong University School of Medicine, Shanghai, China; ^3^ Key Laboratory of Cell Differentiation and Apoptosis of Chinese Ministry of Education, Department of Pathophysiology, Shanghai Jiao Tong University School of Medicine (SJTU-SM), Shanghai, China

**Keywords:** low-level laser therapy, microgravity, wound healing, CCR2, PI3K

## Abstract

**Background and Purpose:**

The risk of skin injuries in space is increasing with longer space missions and a growing astronaut population. This highlights the importance of understanding the adverse effects of weightlessness on wound healing. The objective of this research was to examine the therapeutic potential of Low-Level Light Therapy (LLLT) on skin healing processes under simulated microgravity (SMG) conditions and uncover the underlying molecular mechanisms, thus providing innovative solutions and a sound theoretical basis for space skin injuries.

**Methods:**

Hindlimb unloading (HU) mice models were used to simulate weightlessness conditions, with or without a complete management of LLLT for 14 days. A systematic testing consisting of HE, Masson and immunohistochemical staining was performed against the standardized mouse tissue specimens. *In vitro* assessment of cellular biological functions under SMG conditions was carried out in the rotation system of culture (RSOC) using HaCaT and NIH3T3 cell-lines.

**Results:**

Under SMG conditions, LLLT significantly reduced skin wound area in HU mice, especially on Days 10 (p < 0.001), accompanied by increased collagen deposition and elevated levels of Ki67 and CD31. Moreover, LLLT showed impressive anti-inflammatory effects represented by the reduced in pro-inflammatory markers including LY6G, F4/80 and CD86, as well as the decreased levels of IL-1β, IL-6 and TNF-α. Conversely, an elevation in the anti-inflammatory marker CD206 was observed. By employing bioinformatics technology, we further found the PI3K/AKT signaling was prominent in the KEGG pathway analysis and CCR2 acted as a hub gene in the interaction network. Therefore, we demonstrated that LLLT could enhance the phosphorylation of PI3K/AKT and reduce CCR2 expression under SMG conditions, while CCR2 knockdown promoted the phosphorylation of PI3K/AKT, suggesting an important role of CCR2/PI3K/AKT signal axis in LLLT-accelerated wound healing under SMG conditions.

**Conclusion:**

LLLT induced activation of the PI3K/AKT signaling pathway through suppression of CCR2 expression, which significantly enhanced skin wound healing under SMG conditions.s.

## 1 Introduction

With the increasing frequency of space missions, microgravity has emerged as a significant concern for the health and wellbeing of astronauts. One particular issue of concern is the impairment of cutaneous wound healing under microgravity conditions (Nguyen et al., 2021; Riwaldt et al., 2021). Skin wound healing involves a diverse array of cell types, including epidermal cells, fibroblasts, immune cells and vascular endothelial cells, as well as cytokines such as transforming growth factor β, vascular endothelial growth factor, epidermal growth factor and fibroblast growth factor, which collaborate to coordinate the repair and regeneration of wounds. The absence of gravitational force results in alterations in fluid distribution, immune function and inflammation, affecting the morphology, function and signal transduction of the relevant cells, leading to delayed wound healing and increased susceptibility to infections (Demontis et al., 2017; Adamopoulos et al., 2021).

Research has shown that LLLT can enhance wound healing under normal gravity conditions (Dompe et al., 2020; Leyane et al., 2021). LLLT utilizes specific wavelengths of light to stimulate cellular metabolism, reduce inflammation and enhance collagen synthesis, thereby accelerating the healing process (Avci et al., 2013; Tam et al., 2020). The non-invasive nature and minimal side effects of LLLT make it an attractive therapeutic option for various clinical applications. However, the alterations in cellular biological processes induced by microgravity may impact the interaction between LLLT and cells, subsequently affecting the regulatory effects of LLLT on cellular function and disrupting the wound repair process. Currently, knowledge in this area is relatively limited. Therefore, further research is necessary to investigate the impacts and mechanisms of LLLT on cutaneous wound healing under microgravity conditions.

The phosphatidylinositol 3-kinase/protein kinase B (PI3K/AKT) signaling pathway plays a critical role in the wound repair mechanism, significantly influencing cell growth, differentiation, migration, blood vessel formation, and metabolisms (Westin, 2014; Liao et al., 2018; Teng et al., 2021). Studies have shown that the activation of C-C chemokine receptor type 2 (CCR2), a chemokine receptor, can result in the activation of the PI3K/AKT signaling pathway, possibly by influencing the recruitment, migration and localization of inflammatory cells, including macrophages and monocytes, thus regulating wound healing (Hao et al., 2020; Xu et al., 2021; He et al., 2023). Additionally, blocking of CCR2 reduced neuroinflammation and neuronal apoptosis through the PI3K/AKT pathway (Rodero et al., 2014; Boniakowski et al., 2018). LLLT was also involved in to endothelial cell proliferation and migration by activating PI3K/AKT signaling pathway, further regulating angiogenesis (Li Y. et al., 2020). However, it is currently unclear whether CCR2/PI3K/AKT contributes to the healing of cutaneous wounds in weightlessness and whether LLLT treatment affects this pathway.

The objective of this research is to investigate the role and underlying mechanism of LLLT in promoting cutaneous wound healing under microgravity conditions. By understanding how LLLT influences wound healing processes in space, we aim to contribute valuable insights to the development of advanced wound management strategies for future space explorers and potentially improve wound healing therapies for patients on Earth with conditions that mimic aspects of microgravity-induced healing impairments.

## 2 Materials and methods

### 2.1 HU rodent model

The experiments were conducted using 84 male C57BL/6J mice that were six to 8 weeks old, from Shanghai Animal Center Laboratory of the Chinese Academy of Sciences. The breeding and experimental protocols received authorization from the Experiment Committee and Animal Care at the School of Medicine, Shanghai Jiao Tong University. The Approval ID is SH9H-2023-A915-1.

After 7 days of environmental acclimation, the rodent HU model was established using previously established methods. Briefly, mouse tails were attachedto a pivoting suspension mechanism located at the top of a specially constructed enclosure measuring 20 cm in length, width, and height. The tails, which were disinfected with 75% ethanol, were attached with a special rubber tube designed for tails, positioning the mice’s hind limbs at a 30° downward angle away from the bottom of the cage, thereby unloading the hind limbs. Control micewere fed under normal gravity. They were raised in their cages and provided tap water and chow for feeding. This system is stable and reliable when creating a weightless rodent model in a simulated space condition in this study.

### 2.2 Creation of wound in HU rodent model

**FIGURE 1:**
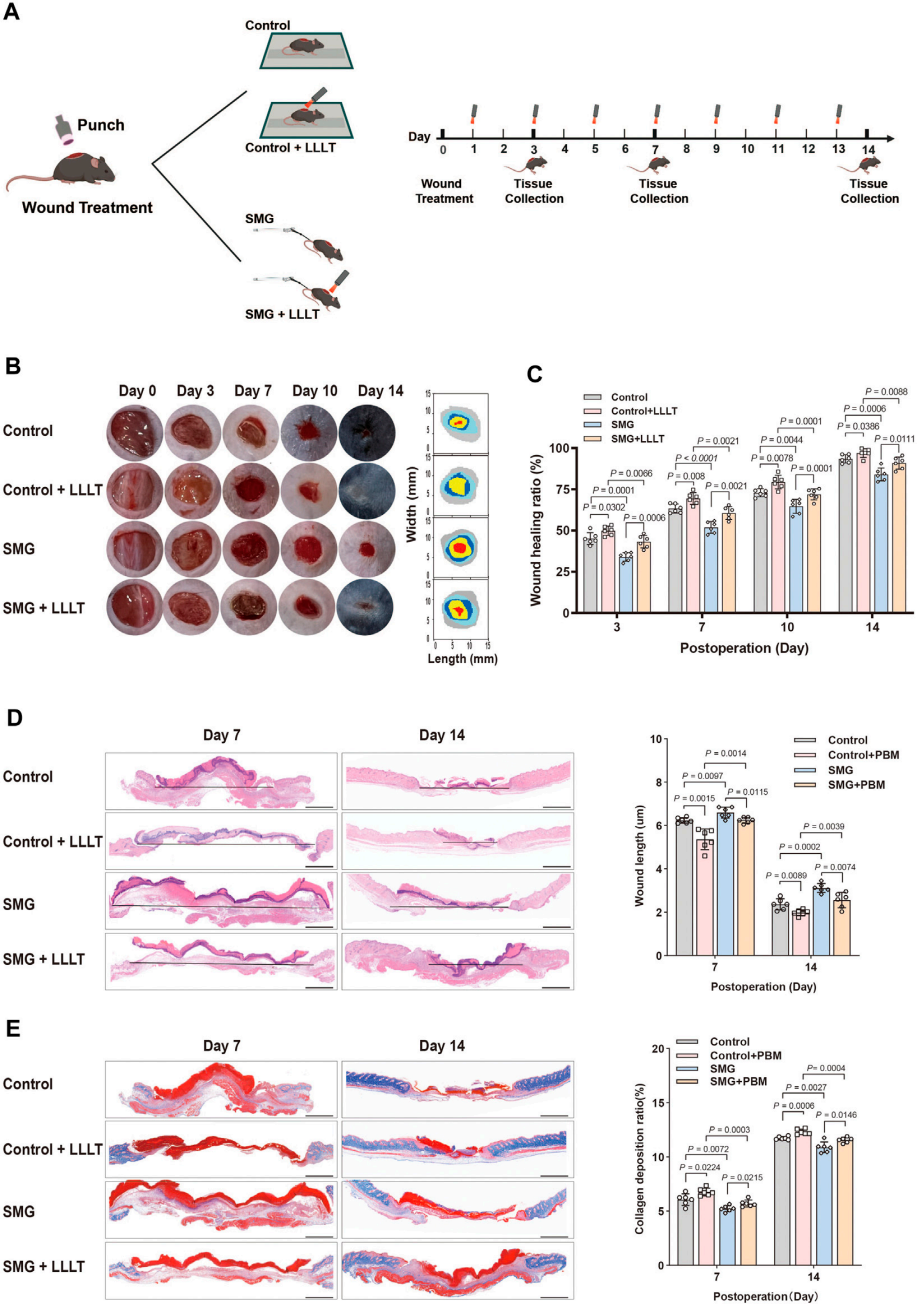
Effects of LLLT on wound healing in the HU mice model. **(A)** Schematic illustration of LLLT therapy for treating the HU model in mice. **(B)** Representative images of the wound healing behavior and dynamic wound healing process on days 0, 3, 7, 10, and 14. **(C)** The wound healing ratio on days 0, 3, 7, 10, and 14. (n = 6). **(D)** HE staining of the wound tissue on days 7 and 14. The granulation tissue thickness in wound tissue on days 7 and 14 (n = 6). **(E)** Masson staining of the wound tissue on days 7 and 14. The collagen deposition ratio of wound tissue on days 7 and 14 (n = 6). Scale bar = 100 μm. The following comparisons revealed significant differences.

After anesthesia with intraperitoneal injection sodium pentobarbital (0.3%, 0.22 mL/10 g), the dorsal area of the rodent was shaved. A full-thickness excisional skin wound with a diameter of 1 cm was created on the dorsal skin of both hindlimb-unloaded (HU) and control mice using a 1 cm round biopsy punch. The depth of the wounds was full thickness skin defect. Wounds were left undressed and kept open throughout the entire experiment. Figure 1A illustrates the study design.

### 2.3 LLLT treatment

Animals were illuminated with prospectively allocated doses of the 632.8 nm laser after being wounded, as depicted in Figure 1A, which outlines the experimental arrangement. The laser utilized was the Laserpulse (LJL40-HA; Shanghai Well Medical Technology Co., Ltd.), with a wavelength range of 640 nm ± 20 nm, adjustable laser power of 0–10 W, adjustable spot diameter of 35–50 mm (5 cm away from the window), and parameters specified in Table 1. Following 24 h of operation, the Control + LLLT group and SMG + LLLT group were subjected to HeNe laser (632.8 nm, 40 mW/cm^2^) for 3.33 min under light-deprived conditions, achieving fluences of 8 J/cm^2^. The control group received white light exposure without any thermal or stimulatory effects. Figure 1A provides an overview of the experiment’s timeline.

**TABLE 1:** Parameters for LLLT treatment.

	In mice	In cells
Wave length (nm)	632.8	632.8
Working mode	CW	CW
Power output (mW)	40	40
Sessions	1	1
Distance to mice/cells (cm)	10	10
Measurement area on target (cm^2^)	1	10
Irradiance (mW/cm^2^)	40	4
Exposure time (min)	3.33	0.5
Energy density (J/cm^2^)	8	1.2
Sum of radiant energy (J)	8	12

Abbreviation: CW, continuous wave.

### 2.4 Grouping and Administration

84 mice were randomly allocated into four groups (n = 18 per group): Control, Control + LLLT, SMG, and SMG + LLLT. The Control group received standard treatment. After establishing the hindlimb unloading (SMG) model, the Control + LLLT and SMG + LLLT groups underwent alternate-day sessions of 8 J/cm^2^ LLLT (632.8 nm, 40 mW/cm^2^) for 2 weeks. Unexpectedly, 12 animals died during anesthesia or operation procedures during the study.

### 2.5 Analysis of wound closure

Photographs of the wounds were taken at intervals of 0, 3, 7, 10 and 14 days after surgery. All images were captured by a Nikon D7500 in a standardized setting to ensure consistent lighting, a fixed height of 30 cm above the wound and to minimize variability. These photographs were uploaded into the ImageJ software for analysis to evaluate the progression of wound healing. To calculate the wound closure percentage, we used the equation: (wound area at baseline—wound area on day A)/wound area at baseline x 100%. In this context, “wound area at baseline” refers to the size of the wound immediately after surgery, whereas “area on day A” refers to the size of the wound on a later day.

### 2.6 Histological examination and immunostaining

A total of 72 mice were sacrificed on days 3 (n = 6), 7 (n = 6), and 14 (n = 6), respectively, and the wound tissues were collected for the experiments. For histopathological analysis, wound tissues from mice were collected on day 7 and 14, fixed with 4% paraformaldehyde, embedded in paraffin, and sectioned into 5-μm thick sections. Tissue sections attached to the slides were deparaffinized, rehydrated, and stained with hematoxylin & eosin, and Masson’s trichrome. For immunofluorescence staining, the sections were deparaffinized and rehydrated, followed by antigen retrieval in 10 mM citrate buffer (pH 6.0) at 98°C for 10 min. The sections were permeabilized for 10 min, blocked with 10% goat serum for 1 h, followed by 5% BSA. Primary antibodies were incubated overnight at 4°C, followed by visual detection with secondary antibodies in blocking buffer for 1 h at room temperature. Immunofluorescence double staining targeted markers including F4/80 (28463-1-AP), CD206 (ab300621), CD86 (ab119857), LY6G (ab238132), KI67 (ab15580), and CD31 (ab222783). Imaging was performed using a DMI 6000B microscope (Leica, Germany), and ImageJ (National Institutes of Health, United States) was used for image analysis and quantification.

### 2.7 Enzyme-linked immunosorbent assay (ELISA)

Tissue samples were dissected on ice, frozen, pulverized, homogenized with extraction buffer, centrifuged, and stored at −80°C before ELISA. Skin tissue samples (0.5 cm in both length and width and extending to a depth beyond the periosteum) from mouse wounds on day 7 were immediately frozen and pulverized (≥5 mg). After addition of extraction buffer with protease inhibitors, samples were homogenized, centrifuged (12,000 g, 10 min, 4°C), and stored at −80°C. Thawed supernatants were mixed prior to ELISA. Concentrations of IL-1β, IL-6, and TNF-α were determined using specific ELISA kits for IL-1β (F10770), IL-6 (F10830), and TNF-α (F11630). These ELISA kits were purchased from Xitang Biological Technology Co., Ltd. in Shanghai, China, and were used according to the manufacturer’s instructions. A microplate reader was used to read the absorbance values at a wavelength of 450 nm.

### 2.8 Cell culture

HaCaT keratinocytes and NIH3T3 murine fibroblasts were cultured in DMEM supplemented with 10% FBS, together with penicillin at a concentration of 100 units/mL and streptomycin at 100 μg/mL. The cells were kept in a controlled environment at 37°C with 5% CO2. The cell cultures used in our experiments were between passages four to five and were subcultured at 80%–90% confluence.

### 2.9 Microgravity simulation

The Rotation System of Culture (RSOC), developed by NASA and manufactured by GeninTech (GeninTech Co., Ltd, Suzhou, China), enables uniform rotation of the High Aspect Ratio Vessel (HARV) and cell growth medium through vertical rotation, creating a low-shear stress environment for three-dimensional (3D) cell culture to effectively simulate microgravity. Prior to exposure to microgravity, the cell samples were composed of well-cultured cells at a density of 3 × 10⁵ cells per ml. Cytodex1 microcarriers (GE17-0485-01, Sigma) were prepared to enhance HaCaT and NIH3T3 cell attachment under microgravity conditions by providing a matrix for cell adhesion, nutrients, physical support, and promoting differentiation of adherent growing cells. These microcarriers were also injected into HARV and T-25 flasks for static control under terrestrial gravity (1 g). The optimal rotation speed of 15 rpm, as recommended by the manufacturer (Frith et al., 2010; Kang et al., 2015; Ebnerasuly et al., 2017), was used for various cell types, including NIH3T3 and HaCaT. The experiment was conducted over 48 h in a cell culture incubator set up to simulate microgravity conditions. For comparison, cells were also cultured in identical containers under static conditions for the same period, ensuring that all other culture conditions were consistent except for the gravitational environment.

### 2.10 Cell wound healing assay and viability

The study investigated how LLLT affected the migration and proliferation of HaCaT and NIH3T3 cells under both standard and simulated microgravity (SMG) conditions. Cells were allocated into 4 experimental setups: (1) Control group: standard gravity; Control + LLLT group: under standard gravity then received LLLT treatment (632.8 nm, 40 mW, 1.2 J/cm^2^ for 0.5 min); (3) SMG group: under SMG; (4) SMG + LLLT group: under SMG then received LLLT treatment. The irradiation time was 0.5 min, with an energy density of 1.2 J/cm^2^ and a total energy of 12 J. Laser treatment was performed 24 h after modeling, with cells adhered to Cytodex. The RSOC apparatus was stopped for irradiation and then returned to microgravity. After treatment, cells were cultured on the RSOC for 24 h before harvesting for the experiment. Cytodex-cell complexes were washed, treated with EDTA-trypsin, and detached by beating. Detached cells were filtered, centrifuged and collected for further processing. Standard scratch assay procedures were followed. After 24 h of simulated microgravity, cells were seeded and grown to confluence. LLLT treatment was then administered and the scratch assay was initiated immediately thereafter. Cell migration was observed by evaluating the wounds initially and after 48 h using an Olympus microscope and quantified using ImageJ software. Concurrently, the proliferation of cells cultured on nanofiber matrices was assessed by Cell Counting Kit-8 (CCK-8, Dojindo, Kumamoto, Japan) assays at specific intervals according to the manufacturer’s guidelines. A mixture of CCK-8 and media was incubated for 2 h at 37°C without exposure to light and the absorbance was measured at 450 nm.

### 2.11 RNA extraction by RT-qPCR analysis

RNA was isolated from the skin of mice treated with or without LLLT using Trizol reagent (15596018CN, Invitrogen) following the specified protocol. The cDNA was synthesized from the supplied RNA using the First-Strand Synthesis System (2680A, Takara) according to the manufacturer’s instructions. Quantitative real-time PCR analysis was performed using SYBR Premix Ex Taq II (DRR081A, Takara) on a 7,500 Real-Time PCR System (Applied Biosystems). Expression levels of specific genes were determined relative to GAPDH (for internal reference) using the 2^−ΔΔCT^ approach to calculate. See Table 2 for details of the primer sequences used.

**TABLE 2:** Sequences of primers for RT-qPCR validation.

Gene name	Forward and reserve primer (5′–3′)
CXCL9	F: CCTAGTGATAAGGAATGCACGATG R: CTAGGCAGGTTTGATCTCCGTTC
CCR2	F: GCTGTGTTTGCCTCTCTACCAG R: CAAGTAGAGGCAGGATCAGGCT
STAT1 GADPH	F: GCCTCTCATTGTCACCGAAGAAC R: TGGCTGACGTTGGAGATCACCA F: TCACCATCTTCCAGGAGCGAGAC R: TGAGCCCTTCCACAATGCCAAAG

### 2.12 RNA-sequence and data processing

RNA was isolated from mouse skin samples using a homogenizer followed by stabilization in RNA later™ solution. The RNeasy MinElute Cleanup Kit was used for RNA extraction to ensure sample quality. The integrity and quantity of total RNA was assessed using the Agilent 4,200 Bioanalyzer and NanoDrop (Thermo Scientific), respectively. Sequencing libraries were prepared using 500 ng of RNA, ensuring an OD260/280 ratio between 1.9 and 2.0 and a minimum RIN value of 8. The ABclonal mRNA-seq Library Preparation Kit was used for paired-end library preparation, and sequencing was performed on the Illumina Novaseq 6,000, generating paired-end reads of 150 bp in length.

Raw sequencing data were processed using in-house perl scripts, involving adapter removal and filtering out low-quality reads (defined as those with a string quality value less than or equal to 25, accounting for more than 60% of the entire reading) and N (base information undetermined) ratio greater than 5%. The resulting clean reads were used for subsequent analyses. For quantification of gene expression levels, Feature Counts were employed to count reads mapped to each gene, and FPKM values were calculated. Differential expression analysis was conducted using the DESeq2 package, with the identification of Differentially Expressed Genes (DEGs) based on adjusted p-values (Bonferroni correction) and fold change criteria (>|2|). A Venn plot was utilized to visualize common DEGs. Enrichment analysis included Gene Ontology (GO) and Kyoto Encyclopedia of Genes and Genomes (KEGG) pathways using the cluster Profiler R package. Gene set enrichment analysis (GSEA) utilized Molecular Signature Database annotations, with significant terms determined at a threshold of p < 0.05. Protein-protein interaction (PPI) network construction using the STRING database, and Cytoscape with the MCODE plugin was employed to identify core genes. Subnetwork analysis was performed with specific criteria, and the statistical analysis of RNA-seq data was conducted in R (version 4.3.0).

### 2.13 Establishment of CCR2 KO cell lines by CRISPR-Cas9

Cells with knocked-down CCR2 expression were generated by selecting three highly effective target sequences from the CCR2 gene using a specialized knockout guide design platform (https://design.synthego.com/#/). The selected oligonucleotides were then combined and integrated into the lentiCRISPR v2 vector by Golden Gate cloning to facilitate expression of the guide RNAs. Validation of the lentiCRISPR v2 sgRNA construct was performed by PCR using specific check F/R primers. This validated construct was introduced into HEK293 cells to evaluate the efficiency of the guide RNA by PCR using CCR2-KO F/R primers. The validated plasmid was then introduced into HaCaT cells. Cells showing potential knockout were selected and seeded into 96-well plates at a density of 0.5 cells per well. CCR2 knockout was confirmed by PCR using the same set of primers and further validated by Western blot analysis.

### 2.14 Western blotting

Skin tissues from rodents were processed in RIPA buffer containing phosphatase and protease inhibitors (Beyotime) and cooled on ice. After centrifugation at 12,000 g for 20 min at 4°C, the clear supernatant was used to determine protein concentration by the BCA method. Subsequently, 50 μg of protein was separated by SDS-PAGE and transferred to PVDF membranes (Millipore). The membranes were blocked with TBST mixed with 5% milk for 1 hour at room temperature and the primary antibodies were incubated overnight at 4°C. The dilution for primary antibodies as following: GADPH (1:50,000; proteintech, cat#60004-1-Ig), AKT(1:1,000; Abclonal, cat#A18675), P-AKT (1:1,000; Abclonal, cat#AP0637), PI3K (1:1,000; Abclonal, cat#A0265), P-PI3K(1:1,000; Abclonal, cat#A22487), CCR2 (1:2000; proteintech, cat#16153-1-AP). The next day, after three TBST washes, the membranes were then incubated with a secondary antibody conjugated to horseradish peroxidase for 1 h at room temperature. Protein bands were detected using ECL reagents (GE Healthcare) and their density was quantified with the ImageJ software (National Institutes of Health, Bethesda, MD). Normalize the intensity of the target protein bands to the intensity of a loading control protein band (GAPDH) to account for variations in sample loading and transfer efficiency.

### 2.15 Statistical analysis

Data were expressed as mean ± standard error of the mean (SEM). An unpaired two-tailed Student’s t-test was used to compare differences between the different groups. One-way ANOVA was used for analyses involving more than two groups. Statistical significance was determined using GraphPad Prism 7.0, with a p-value of less than 0.05 considered indicative of significant differences.

## 3 Results

### 3.1 The impact of LLLT on wound repair in the HU mouse model

We established the HU mouse model, as described in previous studies, to simulate microgravity *in vivo*. (Globus and Morey-Holton, 1985). To investigate the potential of LLLT to promote wound healing under microgravity conditions, we established a rodent model of cutaneous wounding HU on day 0 and started LLLT treatments from day 1 onwards. Mice were irradiated with LLLT every 2 days (Figure 1A). The status of the wound site was systematically recorded throughout the repair process on days 0, 3, 7, 10 and 14. The findings indicated that wound healing was significantly delayed in SMG group compared to the control group, with larger residual wound areas observed on days 3 (p < 0.001), 7 (p < 0.001), 10 (p < 0.005), and 14 (p < 0.001). Meanwhile, by day 14, the wounds of the SMG mice remained unhealed (Figure 1B). Interestingly, after LLLT treatment, mice showed improved wound healing in SMG conditions, with significant reductions in wound area on days 3 (p < 0.001), 7 (p < 0.001), 10 (p < 0.001), and 14 (p < 0.01), and almost complete healed by day 14 (Figures 1B,C). Collectively, it was suggested that LLLT significantly promotes wound healing in SMG mice.

Neogenesis of granulation tissue is essential for tissue repair (Deng et al., 2023; Hamblin, 2017). HE staining was applied to observe the recently developed granulation tissue at the wound site (Figure 1D). On days 7 and 14, SMG mice showed reduced epithelial structures and impaired re-epithelialization compared to controls, which was significantly improved by LLLT treatment on days 7 (p < 0.01) and 14 (p < 0.01). Assessment of collagen fibers at the wound site using Masson’s trichrome staining demonstrated that SMG mice had reduced collagen fiber content compared to control mice, and LLLT treatment significantly increased collagen deposition at the wound site (Figure 1E) on days 7 (p < 0.05) and 14 (p < 0.01). These results suggested that LLLT can accelerate granulation tissue formation, improve re-epithelialization, increase collagen deposition, and effectively promote wound healing in SMG mice.

### 3.2 LLLT promoted cell proliferation and angiogenesis under SMG conditions

**FIGURE 2:**
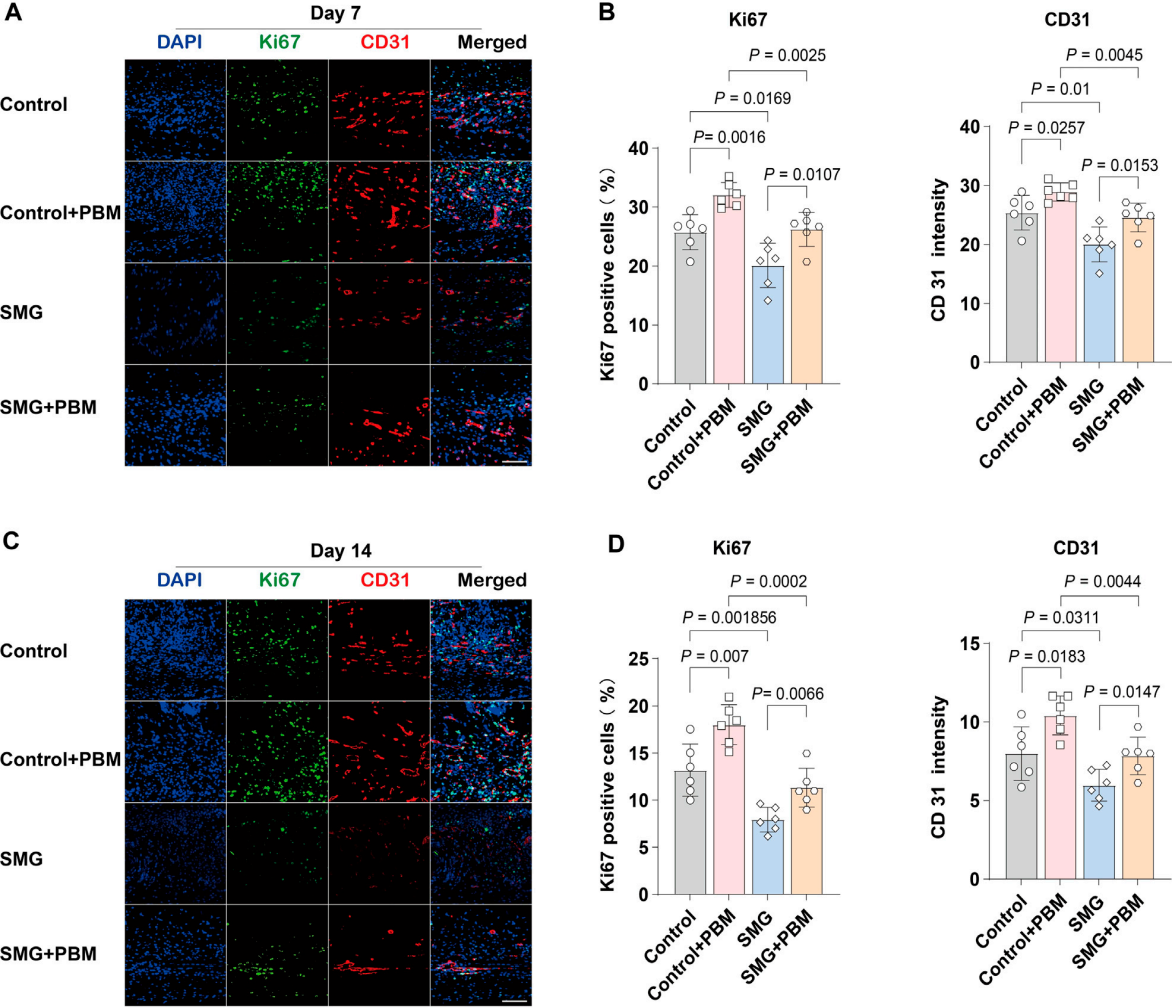
LLLT promoted angiogenesis during the wound healing process under SMG. Immunofluorescence detection of the expression of Ki67 (green) and CD31 (red) in mice skin wounds at **(A)** Day 7 and **(C)** Day 14 (n = 6). Scale bar = 200 μm. Quantification of Ki67 and CD31 area in the wound area at **(B)** Day 7 and **(D)** Day 14.

To investigate the potential mechanisms by which the application of LLLT irradiation affects the generation of granulation tissue cells under SMG conditions, we use immunofluorescence staining to assess the expression of the proliferation marker Ki67 and the endothelial cell marker CD31 at the wound site on both days 7 and 14 after surgery (Yu et al., 2023). As shown in Figures 2A,B, Ki67 expression was lower in SMG group compared with control group, and the application of LLLT treatment markedly increased Ki67 expression. The level of CD31 could be used to assess neovascularization in the renewed tissue (Lee et al., 2015). Consistent with the results of Ki67, application of LLLT treatment significantly increased the expression of CD31 in SMG mice (Figures 2C,D). These results suggested that LLLT may accelerate wound healing in mice under SMG conditions by enhancing cell proliferation, accelerating angiogenesis and promoting granulation tissue formation.

### 3.3 LLLT suppressed tissue inflammation under SMG conditions

**FIGURE 3:**
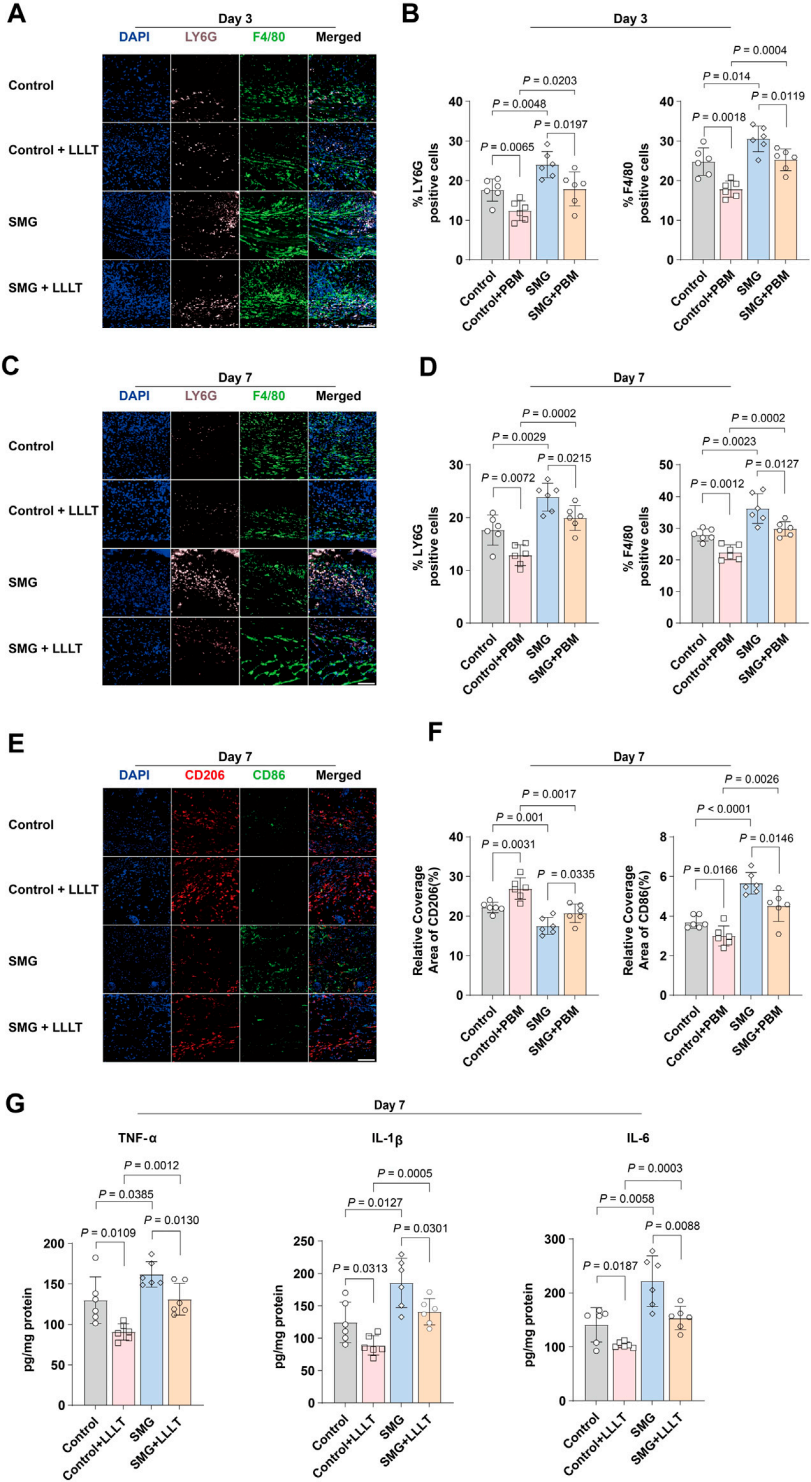
Effects of LLLT in regulating inflammation *in vivo*. Immunofluorescence images of LY6G (pink) and F4/80 (green) in mice skin wound at **(A)** Day 3 and **(C)** Day 7 (n = 6). Scale bar = 200 μm. Quantification of LY6G and F4/80 area in the wound area at **(B)** Day 3 and **(D)** Day 7. **(E)** Representative confocal microscopy images of CD86 (green) and CD206 (red) staining in mice skin wound tissues at Day 7 (n = 6). Scale bar = 200 μm. **(F)** Quantification of CD86 and CD206 area in the wound area at Day 7. Data are presented as mean ± SEM. **(G)** ELISA analysis of IL-1β, IL-6, and TNF-α in mouse skin wound tissues at Day 7 (n = 6). The data were presented as the mean ± standard deviation.

Inflammation is the initial response during typical wound repair (Koh and DiPietro, 2011). To examine the impact of LLLT treatment on inflammation in skin wounds under SMG conditions, immunofluorescence staining was applied to quantify neutrophils and macrophages. LY6G was used to detect infiltrating neutrophils and F4/80 was used to label macrophages (Rose et al., 2012). The findings indicated that the proportions of LY6G^+^ neutrophils and F4/80^+^ macrophages were higher in the wounds of the SMG group than those of the control group on days 3 (Figures 3A,B) and 7 (Figures 3C,D) after injury. LLLT treatment significantly reduced the proportions of neutrophils and macrophages in the SMG mice on days 3 (p < 0.01), and 7 (p < 0.01). M1 macrophages promote early-stage inflammatory response, while M2 macrophages exert an inhibitory effect on inflammation and play a crucial role in promoting tissue repair through the secretion of anti-inflammatory cytokines and facilitation of tissue regeneration. Further study of the expression of CD86 (M1-type macrophages markers) and CD206 (M2-type macrophages markers) (Jaynes et al., 2020; Yu et al., 2022). The results showed that LLLT treatment significantly decreased the expression level of CD86 (p < 0.01) and increased the expression level of CD206 (p < 0.05) in SMG mice (Figures 3E,F). In addition, we also observed the levels of the related pro-inflammatory cytokines IL-1β, IL-6 and TNF-α. The findings indicated that LLLT treatment significantly decreased the expression of pro-inflammatory factors in SMG mice (Figure 3G). These results suggested that LLLT may attenuate inflammation in skin wounds under SMG conditions by inhibiting neutrophil recruitment, suppressing the macrophage polarization towards M1 type, and thus, promoting its polarization towards M2 type Santos et al., 2021).

### 3.4 LLLT promoted the proliferation and migration of HaCaT and NIH3T3 cells

**FIGURE 4:**
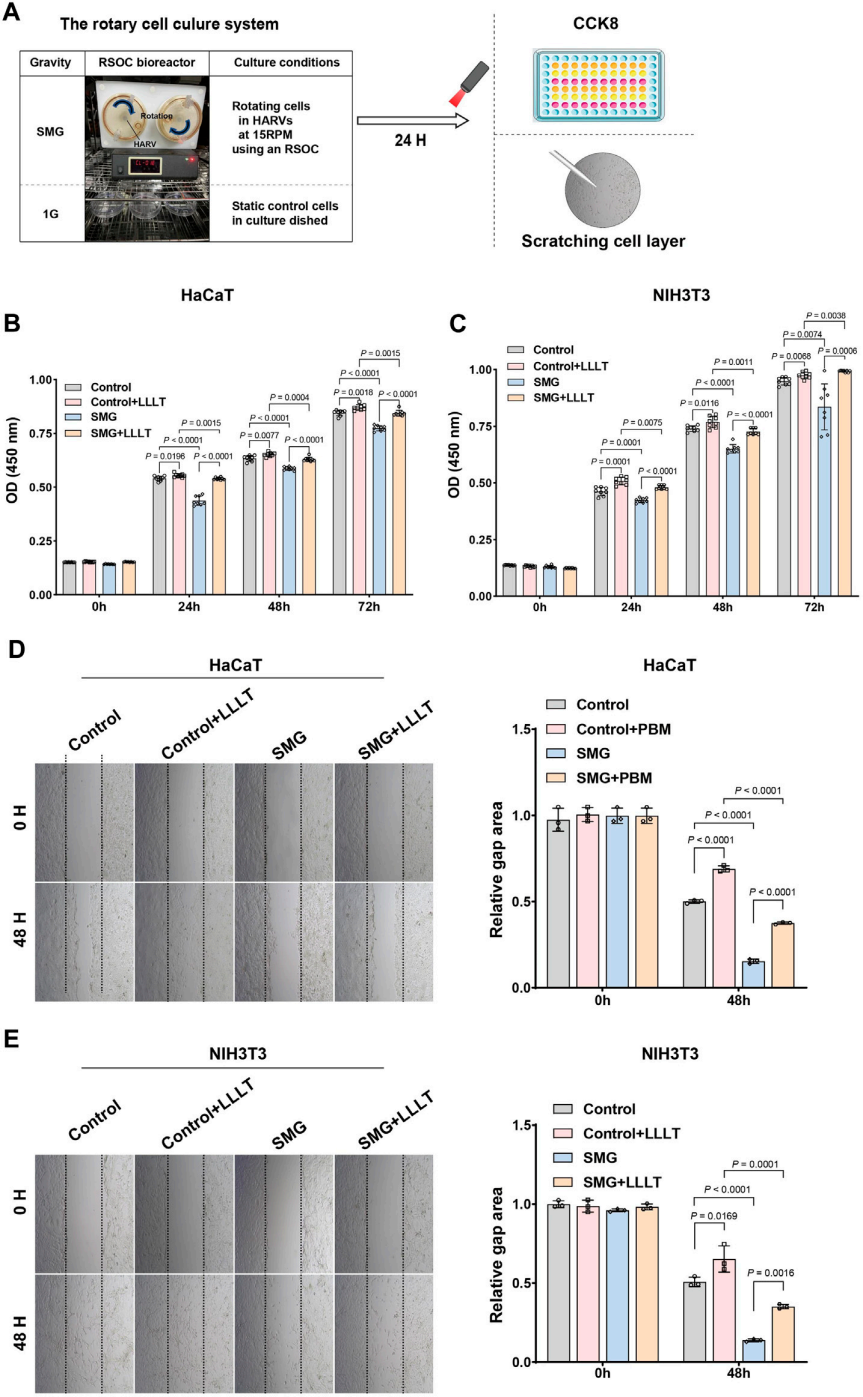
Cell proliferation and migration assay post-microgravity and post-microgravity LLLT. **(A)** Schematic overview of experimental procedure. HaCaT and NIH 3T3 cells were cultured in HARV-RWVs of RSOC to simulate microgravity conditions or in static dishes under normal gravity (1G) controls. Round arrows indicate rotation. During each experiment and post-experiment processing, all steps were performed under similar conditions, including the distribution of the samples in the well plates. The cell viability of **(B)** HaCaT and **(C)** NIH 3T3 cells under SMG treated with LLLT was detected by CCK-8 assay. All experiments were carried out in triplicate. Data are expressed as mean ± standard deviation. Migration of **(D)** HaCaT and **(E)** NIH 3T3 cells under SMG in response to LLLT treatment (40×). Quantification of cells migration rate. Measurement data were expressed as mean ± standard error of mean and analyzed by one-way analysis of variance.

Figure 4A depicted the evaluation of the effect of LLLT treatment on cell proliferation and migration ability under SMG conditions, we conducted *in vitro* experiments by using HaCaT and NIH3T3 cell lines. Our findings of CCK-8 assay indicated that the cell viabilities of both HaCaT and NIH 3T3 cells were significantly decreased in SMG condition, particularly for HaCaT cells at 24H, 48H, and 72H, where the p-values were all less than 0.0001. The application of LLLT rescued the reduction of cell viability (Figures 4B,C). Consistently, the migration ability of both HaCaT and NIH3T3 cells were also reduced in SMG conditions (Figures 4D,E). And LLLT treatment effectively improved the migration ability in SMG conditions. These results indicated that LLLT protected cell proliferation and migration ability under SMG conditions.

### 3.5 LLLT resulted in decreased transcriptomes for inflammation

**FIGURE 5:**
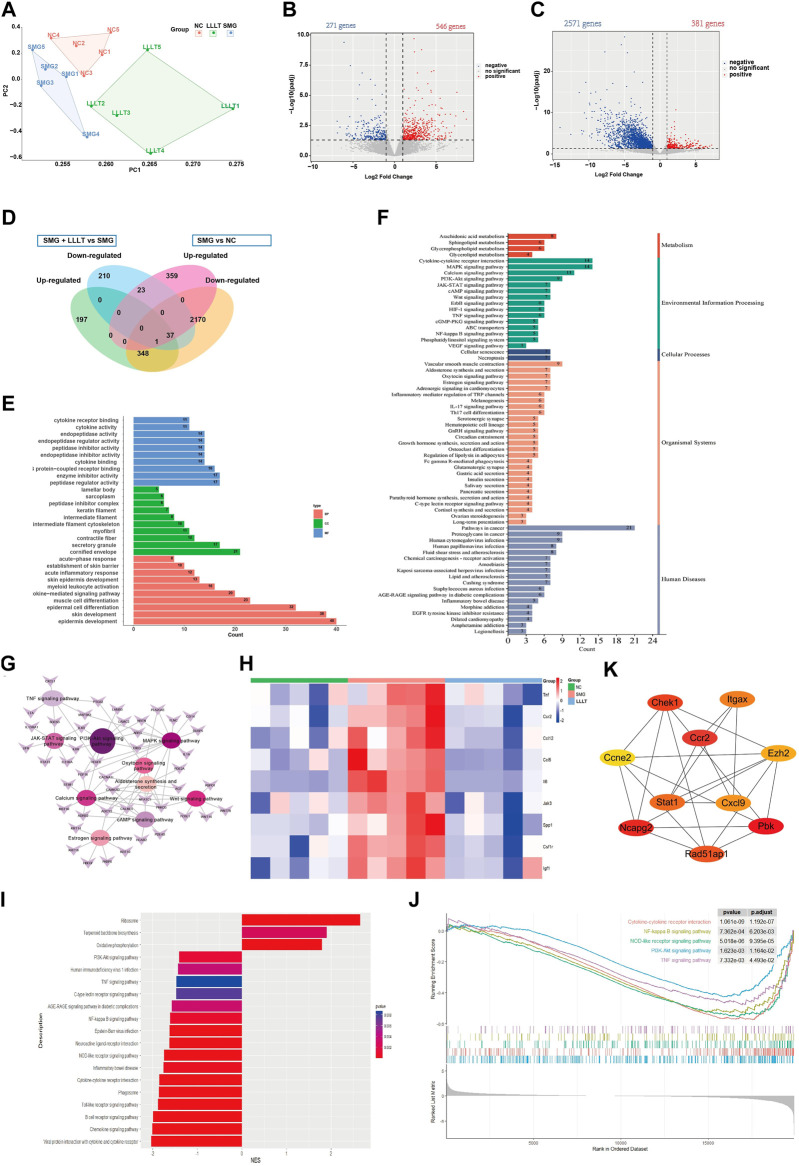
Bioinformatics analysis of DEGs between LLLT and SMG. **(A)** Principle component analysis plot showed separation of Control (NC), SMG and LLLT data by their top two principal components (n = 5). **(B)** Volcano plots of up- and downregulated genes between SMG and Control group. **(C)** Volcano plots of up- and downregulated genes between LLLT and SMG group. **(D)** Venn diagram showing DEGs in the three groups. **(E)** Enriched pathway in GO analysis of Common DEG. BP: Biological process, CC: Cellular component. MF: Molecular function. **(F)** Enriched pathway in KEGG analysis of Common DEG. **(G)** Common DEG-pathway association network diagram. **(H)** The heat map of the inflammatory associated genes expresion level in three groups. **(I)** The Bar plot of GSEA result for LLLT and SMG group. NES >0 represents the activation of this pathway in LLLT group, NES <0 represents the inhibition of this pathway in LLLT group. **(J)** The main inhibited functions of mice in the LLLT group. **(K)** The Cytohubba was used to construct the Top10 hub genes. The figure showed the Top10 hub genes constructed by the MCC method.

To further understand the potential mechanism by which LLLT improves wound healing under microgravity conditions, we conducted a transcriptomic analysis of mice skin wounds using bulk RNA-seq. Seven days after microgravity induced skin damage in mice. The LLLT group was irradiated with LLLT after surgery, once every 2 days (on Day 2, Day 4 and Day 6), and irradiated three times. Wound skin samples were collected on day 1 after the final LLLT treatment for transcriptomic analysis. The NC group (Control group) samples were obtained from normal mice skin tissue at the same wound location as the SMG group. Principal component analysis (Figure 5A) showed significant separation of mRNA transcriptome profiles from the three groups. Following our analysis, we discovered 817 genes exhibiting differential expression (DEGs) between SMG and NC groups, including 546 upregulated genes and 271 downregulated genes (Figure 5B). And 2,952 DEGs was identified between LLLT and SMG, including 381 upregulated genes and 2,571 downregulated genes (Figure 5C). The Venn plot (Figure 5D) shows the overlapping genes between DEGs and further describes the upregulation or downregulation genes of SMG and LLLT. By comparing the DEGs in each analysis between the SMG and LLLT groups, we identified the upregulated genes after SMG treatment and the downregulated genes after LLLT treatment as Common DEGs. Subsequently, we conducted DEGs between the SMG and LLLT groups using GO and KEGG functional enrichment analysis. Our findings revealed enrichment in different functions and pathways among these genes. We found that the primary pathways identified were predominantly associated with inflammatory and immune responses. GO analysis (Figure 5E) showed that common DEGs were most significant in the biological activity of epidermal cells and muscle cells, such as skin development, epidermis development, skin barrier, muscle cell differentiation, skin epidermis development, etc. In terms of immunity, acute phase response, acute inflammatory response, cytokine-mediated signaling pathway, and myeloid leukocyte cell activation. KEGG analysis revealed (Figure 5F) that the common DEGs are primarily associated with immune cell-related signaling pathways. These pathways include PI3K/AKT, MAPK, JAK/STAT, cAMP and Wnt signaling pathways. In the KEGG pathway enrichment network (Figure 5G), the PI3K/AKT pathway occupies a central position. The heat map (Figure 5H) shows the high expression of some common inflammatory factors in the SMG group, such as TNF, CCR2, IL-6. After LLLT treatment, the expression level of these inflammatory genes decreased to varying degrees. LLLT can effectively reduce the inflammatory response after SMG. The GSEA results (Figures 5I,J) showed that after LLLT treatment, Ribosome, Terpenoid backbone biosynthesis, and oxidative physiology were activated, while inflammatory-related pathways such as PI3K/AKT signaling pathway, NOD-like receptor signaling pathway, Toll-like receptor signaling pathway, Chemokine signaling pathway, TNF signaling pathway, NF-κB signaling pathway were inhibited. The above results also indicate that LLLT effectively alleviates the inflammatory response caused by SMG. A protein-protein interaction (PPI) network was constructed for the common DEG using STRING and analyzed using Cytoscape software. Using Cytoscape software based on the CytoHubba MCC algorithm (Figure 5K), identify the ten most significant hub genes: CCR2, PBK, NCAPG2, CHEK1, CXCL9, STAT1, RAD51AP1, ITGAX, CCNE2, and EZH2. CCR2 emerged as the most significantly upregulated gene, achieving the top rank by the MCC technique, and was pinpointed as a central hub gene within the interaction network. Following LLLT treatment after SMG, it was observed that the inflammation induced by SMG was attenuated, suggesting the pivotal involvement of CCR2 as a fundamental hub gene in this process.

### 3.6 CCR2 played a key role in LLLT to accelerate wound healing under SMG conditions

**FIGURE 6:**
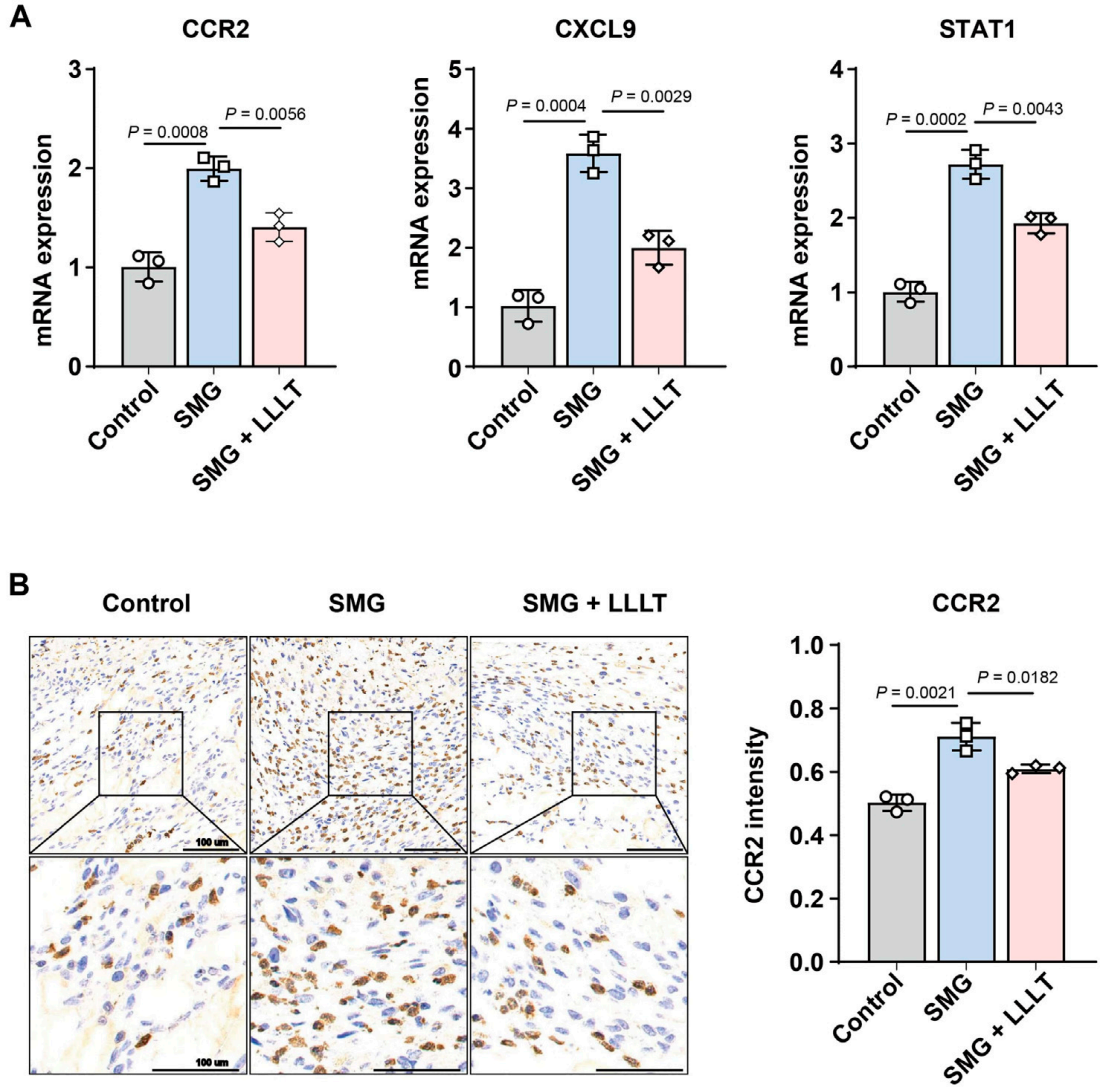
Analyses of potential LLLT-related genes. **(A)** q-RT-PCR analysis of CCR2, CXCL9 and STAT1 mRNA expression level at Day 7 (n = 3). **(B)** Immunohistochemistry analysis and data visualization of CCR2 in mice skin wound at Day 7 (n = 3). The data were presented as the mean ± standard deviation.

Next, we applied qPCR and immunohistochemical staining to validate the transcriptome sequencing results, which were consistent with the transcriptome results that LLLT treatment significantly decreased the expression level of CCR2, CXCL9 and STAT-1 mRNA under SMG conditions. As shown in Figures 6A,B, the expression levels of CCR2 mRNA (p = 0.0008) and protein (p = 0.0021) were significantly increased in the SMG group compared with the control group. Nonetheless, LLLT reduced CCR2 levels. These results suggest that LLLT may inhibit inflammatory responses and enhance cellular proliferation and migration through upregulating CCR2 expression, thus promoting the process of cutaneous wounds healing in the SMG conditions, and the specific mechanism needs to be further elucidated.

### 3.7 The downregulation of CCR2 induced-by LLLT application activates PI3K/AKT pathway

**FIGURE 7:**
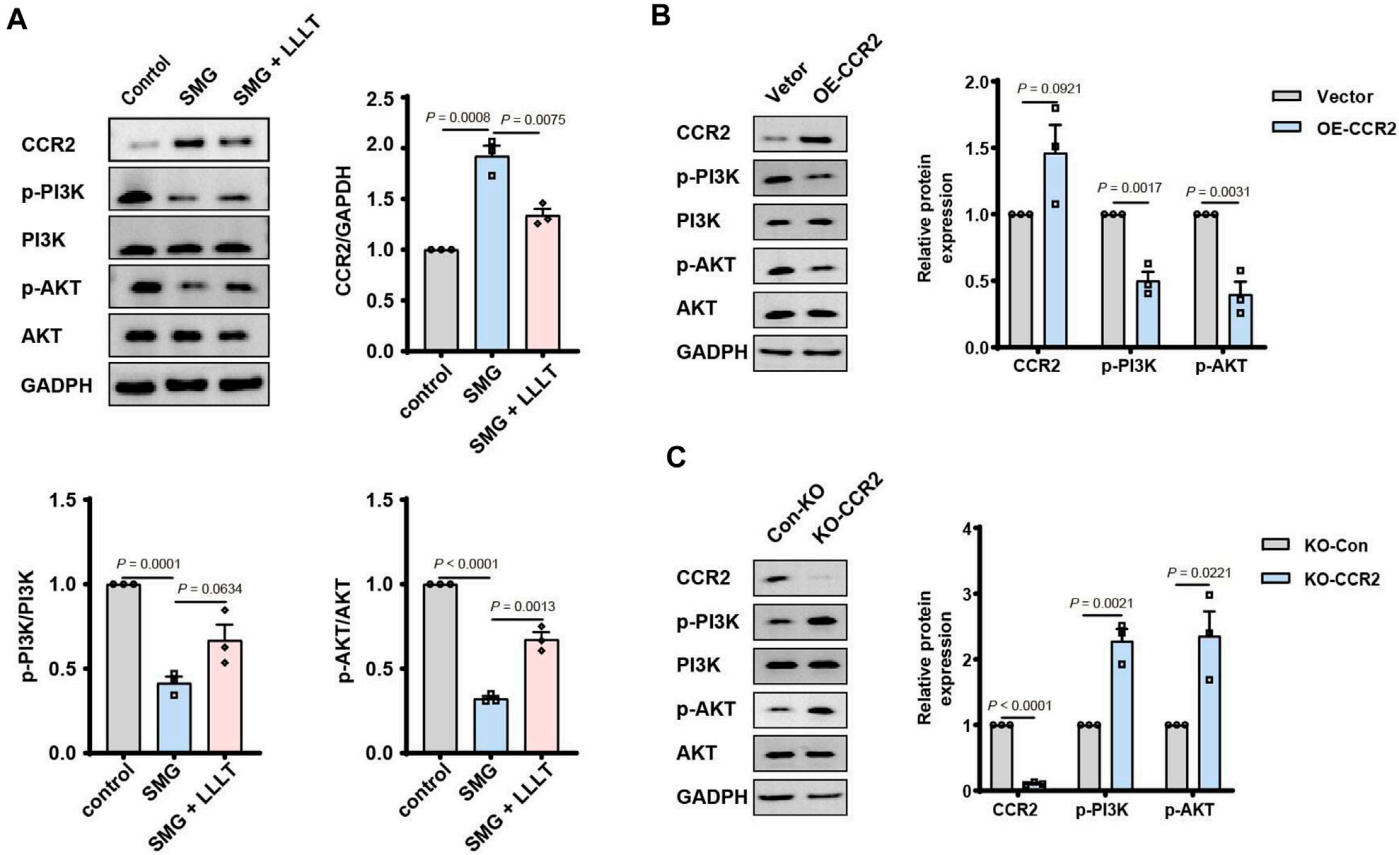
LLLT activated PI3K/AKT signaling pathway via downregulating the level of CCR2 in SMG condition. **(A)** Western blotting of CCR2, p-PI3K, PI3K, p-AKT and AKT in mice skin wound at Day 7 (n = 3). The data were presented as the mean ± standard deviation. **(B)** Western blotting of CCR2, p-PI3K, PI3K, p-AKT and AKT after CCR2 overexpression and knockdown.

To further verify the correlation between CCR2 and PI3K/AKT pathway, the expression of CCR2, P-PI3K, PI3K, P-AKT and AKT in different groups was analyzed by Western blotting. The findings indicated that the expression of CCR2 was negatively correlated to both P-PI3K and P-AKT (Figure 7A). Based on these results, the overexpression and knockdown of CCR2 were performed to confirm whether CCR2 regulated the function of PI3K/AKT pathway as an up-stream molecule. The overexpression of CCR2 inhibited the expression P-PI3K and P-AKT (Figure 7B). Consistently, the knockdown of CCR2 promoted the phosphorylation of PI3K and AKT (Figure 7C). Therefore, we speculated that LLLT rescued the inhibition of PI3K/AKT pathway in SMG condition via downregulating the expression of CCR2.

## 4 Discussion

The process of skin wound healing is intricate and involves multiple cellular and molecular events. In microgravity conditions, the healing process is further complicated due to altered cellular behavior and impaired tissue regeneration (Wilkinson and Hardman, 2020a; Cialdai et al., 2022). LLLT has emerged as a potential therapeutic approach to enhance wound healing. This research examines the role of LLLT in promoting the repair of skin wounds under microgravity conditions and its underlying mechanisms, focusing on the PI3K/AKT signaling pathway. These findings revealed that LLLT significantly enhances wound healing in SMG conditions, as evidenced by improved granulation tissue formation, increased collagen deposition, and decreased inflammatory response.

LLLT has been extensively used in wound treatment, yielding promising results^[^ (Cialdai et al., 2022; Posten et al., 2005)^]^. Research by Calin and Parasca has demonstrated that lasers within the 630–700 nm range possess remarkable abilities for tissue repair (Calin and Parasca, 2010). Consequently, in our study, we opted to utilize a 660-nm laser as an adjunctive therapeutic approach. Granulation tissue formation plays a pivotal role in the healing of wounds, providing a scaffold for cellular infiltration, angiogenesis, and subsequent tissue repair. It is characterized as a temporary extracellular matrix composed of fibroblasts, blood vessels, immune cells, and components of extracellular matrix like collagen and proteoglycans (Li X. et al., 2020). Fibroblasts and angiogenesis are essential factors in the production of collagen and the development of granulation tissue (Wilkinson and Hardman, 2020b). Research suggests that LLLT’s photon energy induces photophysical, photochemical, and photobiological effects (Huang et al., 2009). In addition to stimulating the proliferation of epithelial cells, osteoblasts, and fibroblasts, as well as promoting collagen synthesis, LLLT effectively mitigates edema and hyperemia during inflammatory processes (Oliveira Sierra et al., 2013; Huang et al., 2023). Supporting these findings, our investigation on HU mice treated with LLLT also demonstrated enhanced collagen deposition, increased cell proliferation, and augmented angiogenesis. These observations strongly indicate that LLLT may accelerate skin wound healing under SMG conditions by facilitating the development of granulation tissue.

The proliferation and migration of epithelial cells and fibroblasts are closely linked to the formation of granulation tissue. During granulation tissue formation, epithelial cells move from the wound margins to envolop the injured area, while fibroblasts proliferate and migrate into the wound site, synthesizing and organizing the extracellular matrix. The coordinated actions of these cell types promote the formation of a functional granulation tissue that supports tissue repair, angiogenesis, and the infiltration of immune cells (Nunan et al., 2015; Cheng et al., 2016; Haensel and Dai, 2018; Koike et al., 2020). In our *in vitro* experiments, we employed two commonly used cell lines, HaCaT (human keratinocyte) and NIH3T3 (mouse fibroblast), to examine the effects of LLLT on cell proliferation and migration under SMG conditions. Our research demonstrated that LLLT significantly enhances the proliferation and migration rates of both HaCaT and NIH3T3 cells. The increased proliferation and migration of these cells could contribute to faster re-epithelialization, improved extracellular matrix deposition, and ultimately, more effective wound repair under reduced gravity conditions.

It is well-established that inflammation constitutes the early stages in the overlapping stages of tissue repair (Cheng et al., 2016). Prolonged inflammation has been found to hinder tissue repair and cause excessive scar development (Eming et al., 2007). Macrophages are critical in regulating inflammation and facilitating wound healing. They secrete growth factors such as epidermal growth factor, keratinocyte growth factor and transforming growth factor, which induce fibroblast and keratinocyte proliferation, collagen and extracellular matrix protein production, thereby contributing to granulation tissue development and re-epithelialization (Krzyszczyk et al., 2018). *In vitro* tissue repair studies have categorized macrophages into two distinct phenotypes based on their functions: “classically activated” or M1-type macrophages, which release pro-inflammatory cytokines like IL-12, IL-1β, IL-6, TNF-α and inducible nitric oxide synthase, and are involved in pathogen clearance, inflammatory cytokine release and Th1 response generation; “alternatively activated” or M2-type macrophages, which promote angiogenesis, anti-inflammatory cytokine release and resolution of inflammation (West et al., 2011; Wolf et al., 2021). Our findings revealed that LLLT treatment induces the shift of macrophages towards an M2 phenotype while inhibiting their transition to the M1 phenotype in HU mice. This shift in macrophage polarization is accompanied by reduced skin wound inflammation and accelerated wound healing in HU mice. However, it is crucial to note that the relationship between inflammation and granulation tissue formation is complex and interdependent (Chen et al., 2021). While LLLT appears to mitigate the inflammatory response and enhance wound healing, the precise temporal sequence and interplay between LLLT’s effects on inflammation and granulation tissue formation remain to be elucidated. Further studies are needed to decipher the underlying mechanisms and potential sequential actions of LLLT on inflammation and granulation tissue development, which will provide valuable insights into its therapeutic potential for optimizing wound healing under various conditions.

The PI3K/AKT signaling pathway is a key regulator of a wide variety of cellular processes, including proliferation, differentiation, migration, angiogenesis, and Metabolic processes. Furthermore, it is essential for skin development and homeostasis. In wound healing, this pathway is critically involved, as disrupted AKT signaling can inhibit keratinocyte proliferation and prolong epithelial wound healing. (Xu et al., 2009). The AKT pathway is vital for modulating macrophage survival, migration, and proliferation, as well as coordinating their responses to various metabolic and inflammatory cues (Ding et al., 2012). Studies have shown that during delayed cutaneous wound healing, both tissue remodeling and reduced AKT3 expression are extended. M2 macrophages, which originate from delayed wound tissue, are deficient in AKT3 and fail to promote the proliferation and migration of human skin fibroblasts (Gu et al., 2020). A substantial body of research indicates that imbalances in the PI3K/AKT pathway are frequently linked to various types of skin cancer, including malignant melanoma, basal cell carcinoma, and cutaneous squamous cell carcinoma, and contribute to poor outcomes (Chamcheu et al., 2019). Our findings revealed that LLLT treatment suppresses the activation of the PI3K/AKT signaling pathway in HU mice, which paradoxically promotes wound healing. In addition, numerous findings have concentrated on the significance of CCR2 in cutaneous tissue repair. Rodero and colleagues demonstrated that CCR2-mediated macrophage recruitment occurs in the first 4 hours following tissue injury in a murine scalp excisional wound model. The absence of CCR2 was found to impair wound healing by modulating the inflammatory response. In our research, we observed that LLLT treatment suppresses CCR2 expression in HU mice, thereby promoting wound healing. The inhibition of CCR2 has been shown to alleviate neuroinflammation and neuronal apoptosis following subarachnoid hemorrhage through the PI3K/AKT pathway (Tian et al., 2022). Although our results showed that application of LLLT reduces CCR2 levels and promoted phosphorylation of PI3K and AKT under SMG conditions, the knockdown of CCR2 also promotes phosphorylation of PI3K and AKT. However, it is unclear whether LLLT effects its wound healing effects by acting on CCR2, which subsequently modules the PI3K/AKT pathway, inhibits macrophage recruitment, attenuates inflammation, and ultimately promotes granulation tissue formation, accelerating skin wound healing under SMG conditions. It is necessary to focus on the specific upstream and downstream regulatory mechanisms in future studies. In conclusion, our study demonstrates that LLLT has positive effects on tissue repair processes in SMG conditions by reducing CCR2 expression and activating PI3K/AKT signaling pathway. These findings hold promise for developing novel therapeutic strategies to improve wound healing outcomes in space missions and in terrestrial settings where normal healing is compromised. Nevertheless, A major limitation of our study is the use of the HU mouse model to simulate microgravity, which may not fully replicate the conditions encountered by humans in space. In addition, *in vitro* experiments with HaCaT and NIH3T3 cell lines, while informative, do not encompass the full complexity of human tissue responses. The differences in wound healing between mice and humans further complicate the direct translation of our findings to the clinical setting. Therefore, the parameters of LLLT that were effective in our animal model may need to be adjusted for optimal use in humans.

## Data Availability

The datasets presented in this study can be found in online repositories. The names of the repository/repositories and accession number(s) can be found below: https://www.ncbi.nlm.nih.gov/, CCR2.
